# Supplementary material for: Uridine-derived ribose fuels glucose-restricted pancreatic cancer
Source: Nature. Author manuscript; Available in PMC 2024 Jun 1. (PMC10232363; doi:10.1038/s41586-023-06073-w)
Supplement: Supp Fig11 [file NIHMS1902848-supplement-Supp_Fig11.pptx]

## Slide 1
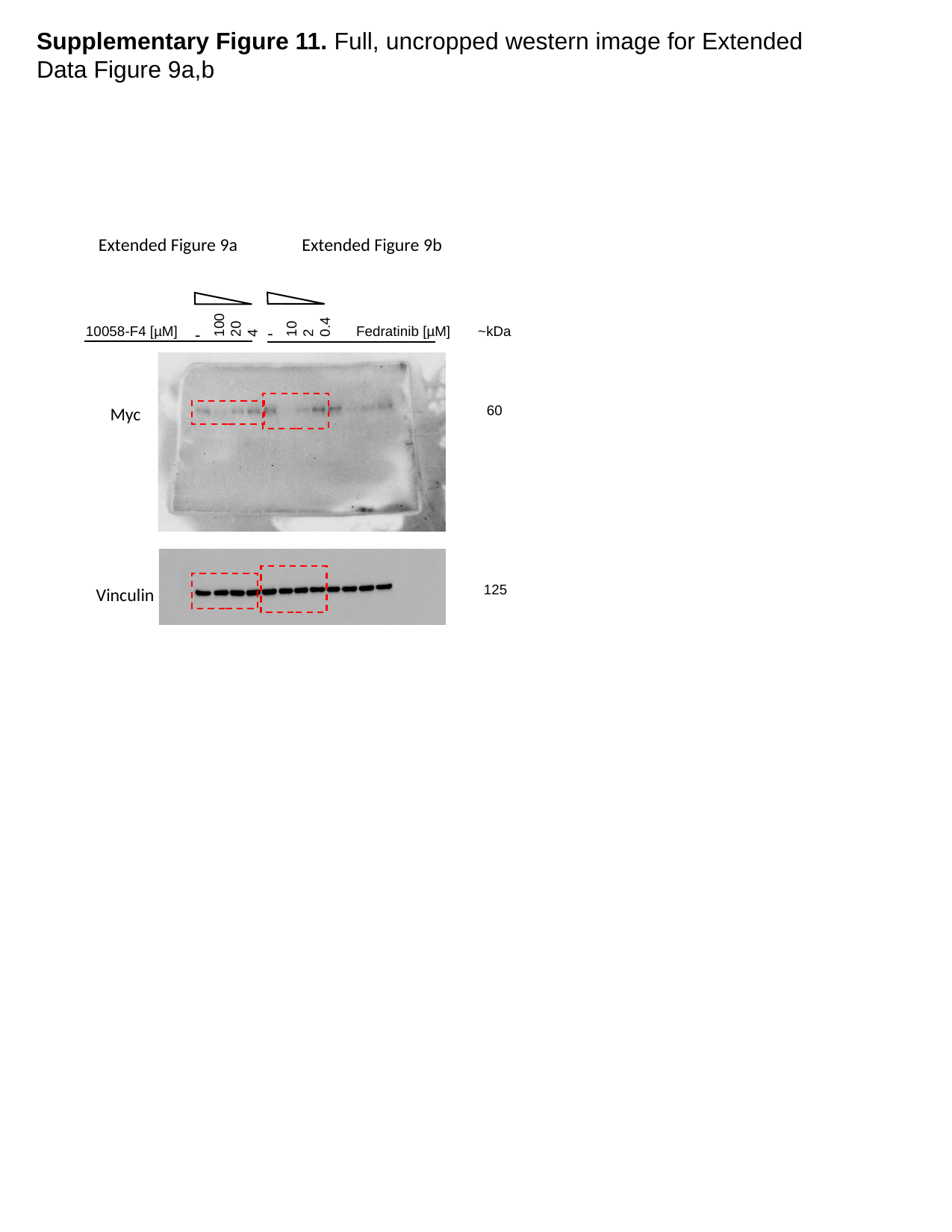

Supplementary Figure 11. Full, uncropped western image for Extended Data Figure 9a,b
Extended Figure 9a
Extended Figure 9b
100
20
4
10
2
0.4
~kDa
Fedratinib [µM]
-
10058-F4 [µM]
-
60
Myc
125
Vinculin
