# Supplementary material for: Uridine-derived ribose fuels glucose-restricted pancreatic cancer
Source: Nature. Author manuscript; Available in PMC 2024 Jun 1. (PMC10232363; doi:10.1038/s41586-023-06073-w)
Supplement: Supp Fig10 [file NIHMS1902848-supplement-Supp_Fig10.pptx]

## Slide 1
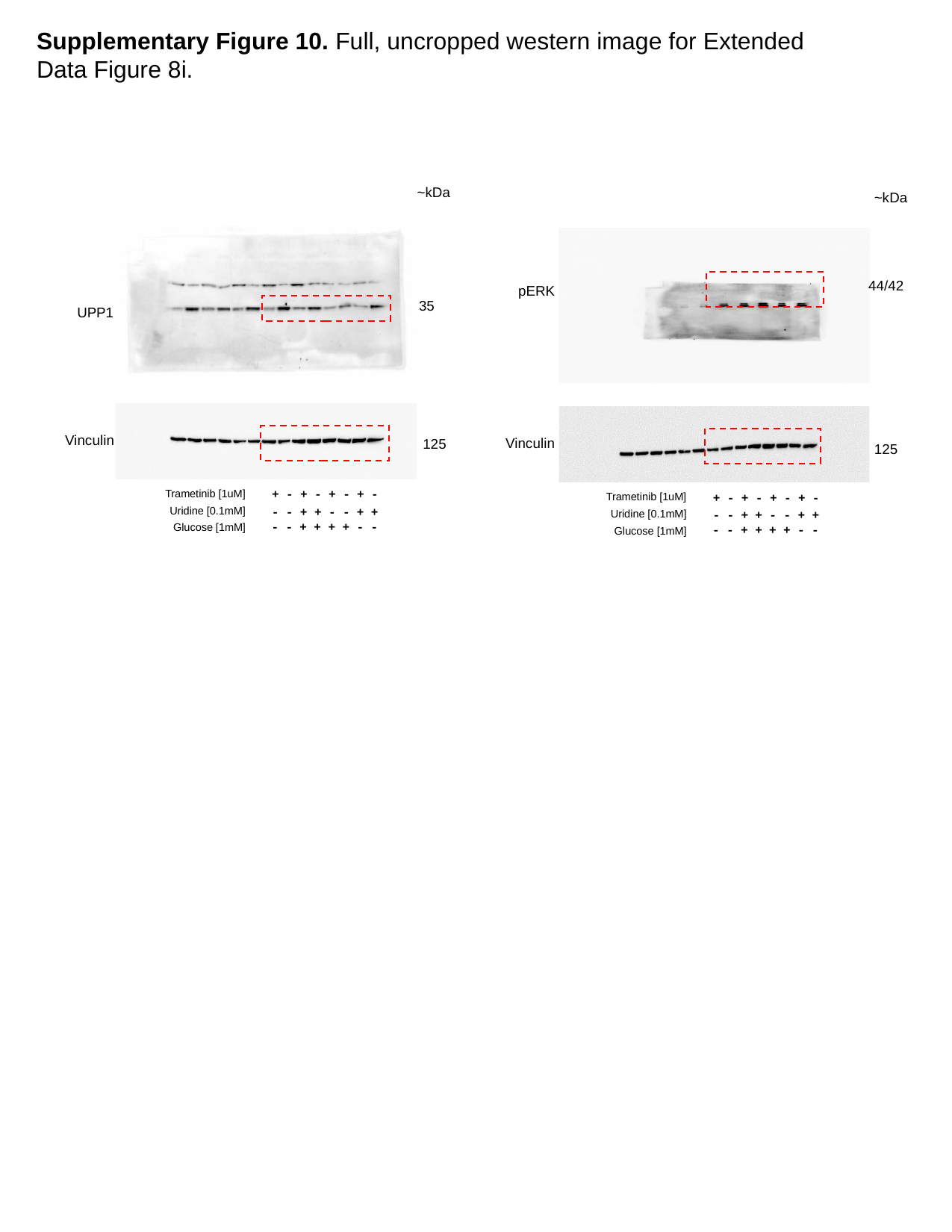

Supplementary Figure 10. Full, uncropped western image for Extended Data Figure 8i.
~kDa
~kDa
44/42
pERK
35
UPP1
Vinculin
Vinculin
125
125
Trametinib [1uM]
+ - + - + - + -
Trametinib [1uM]
+ - + - + - + -
Uridine [0.1mM]
- - + + - - + +
Uridine [0.1mM]
- - + + - - + +
- - + + + + - -
Glucose [1mM]
- - + + + + - -
Glucose [1mM]
