# Supplementary material for: Uridine-derived ribose fuels glucose-restricted pancreatic cancer
Source: Nature. Author manuscript; Available in PMC 2024 Jun 1. (PMC10232363; doi:10.1038/s41586-023-06073-w)
Supplement: Supp Fig9 [file NIHMS1902848-supplement-Supp_Fig9.pptx]

## Slide 1
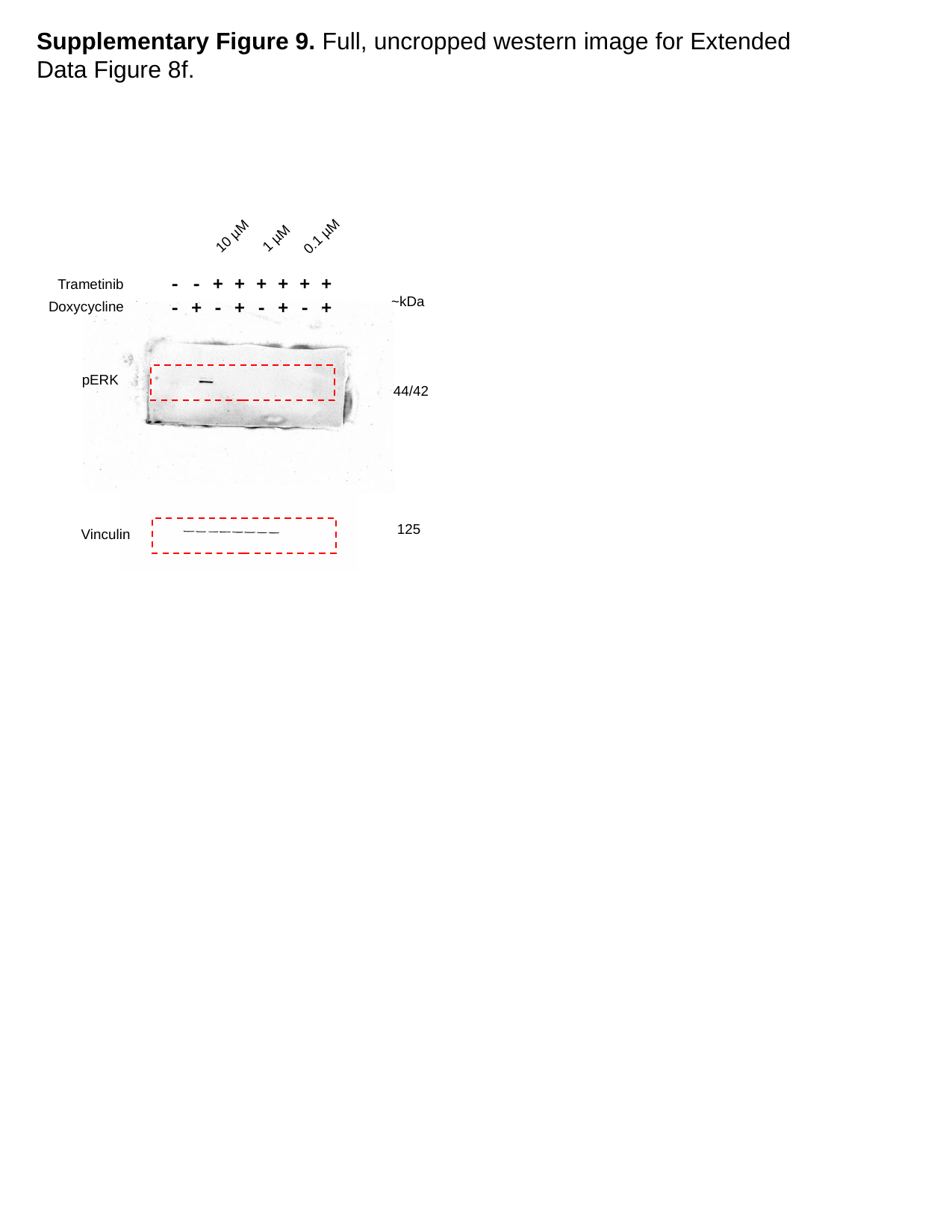

Supplementary Figure 9. Full, uncropped western image for Extended Data Figure 8f.
10 µM
0.1 µM
1 µM
- - + + + + + +
Trametinib
~kDa
- + - + - + - +
Doxycycline
pERK
44/42
125
Vinculin
