# Supplementary material for: Uridine-derived ribose fuels glucose-restricted pancreatic cancer
Source: Nature. Author manuscript; Available in PMC 2024 Jun 1. (PMC10232363; doi:10.1038/s41586-023-06073-w)
Supplement: Supp Fig5 [file NIHMS1902848-supplement-Supp_Fig5.pptx]

## Slide 1
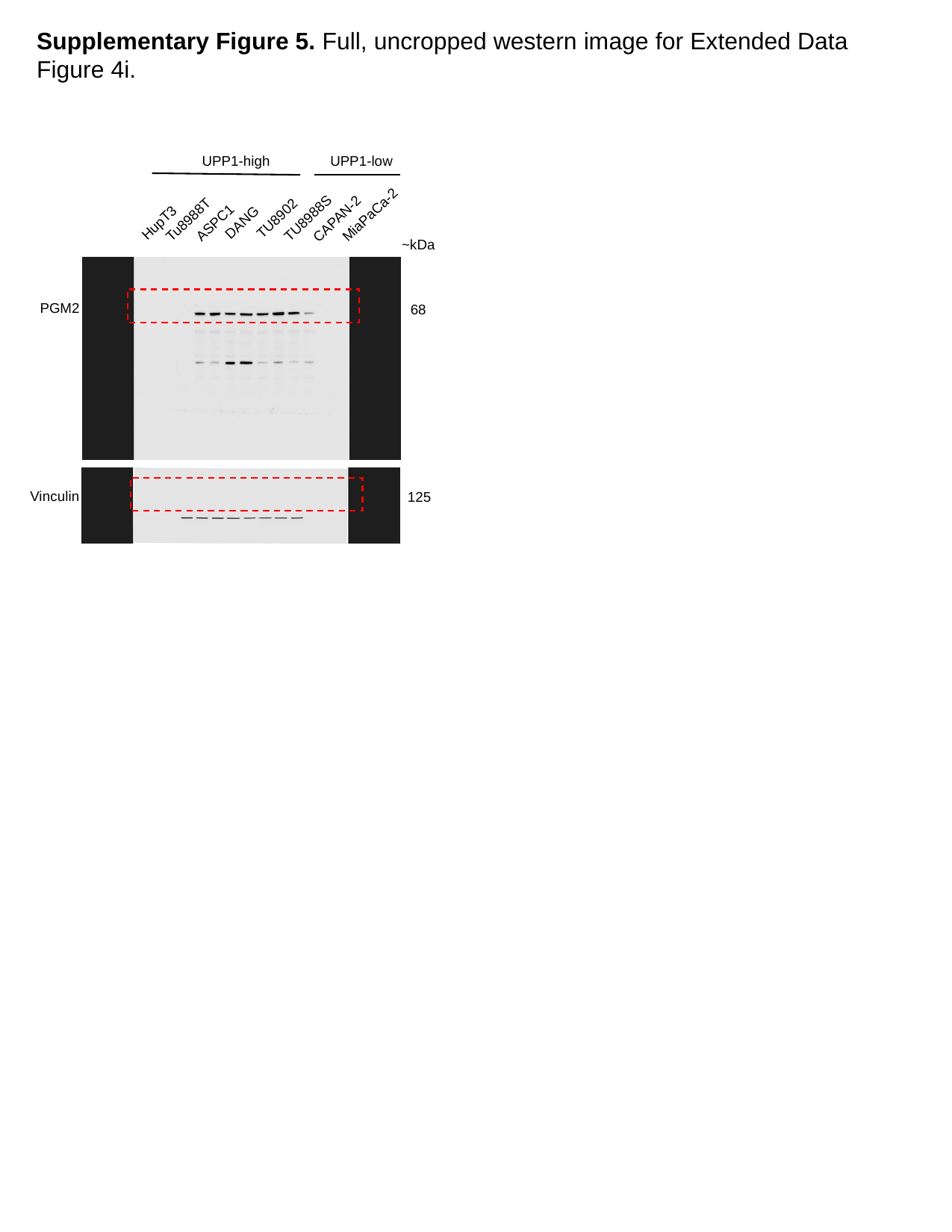

Supplementary Figure 5. Full, uncropped western image for Extended Data Figure 4i.
UPP1-high
UPP1-low
MiaPaCa-2
TU8988S
TU8902
CAPAN-2
Tu8988T
HupT3
DANG
ASPC1
~kDa
PGM2
68
Vinculin
125
