# Supplementary material for: Uridine-derived ribose fuels glucose-restricted pancreatic cancer
Source: Nature. Author manuscript; Available in PMC 2024 Jun 1. (PMC10232363; doi:10.1038/s41586-023-06073-w)
Supplement: Supp Fig6 [file NIHMS1902848-supplement-Supp_Fig6.pptx]

## Slide 1
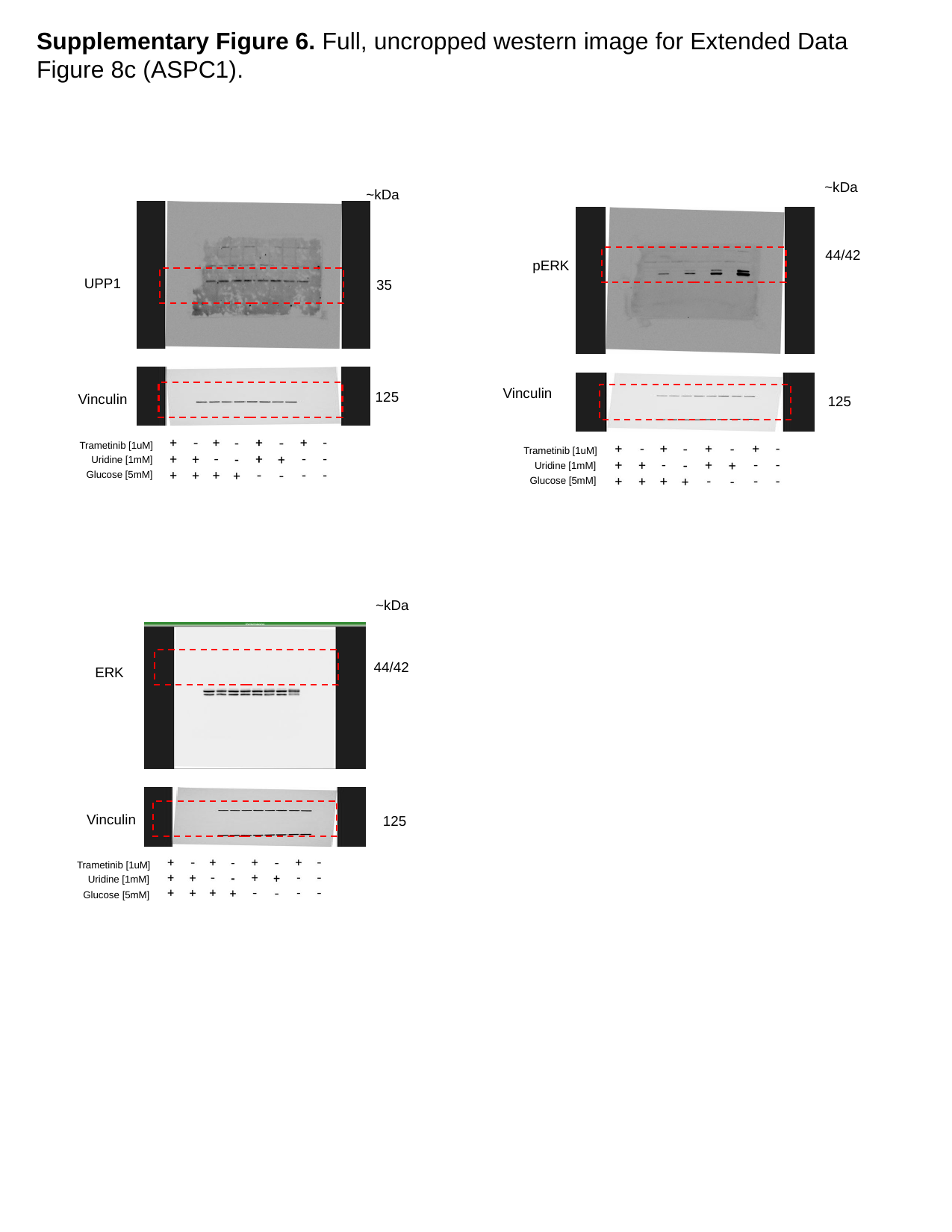

Supplementary Figure 6. Full, uncropped western image for Extended Data Figure 8c (ASPC1).
~kDa
~kDa
44/42
pERK
UPP1
35
Vinculin
125
Vinculin
125
Trametinib [1uM]
Trametinib [1uM]
Uridine [1mM]
Uridine [1mM]
Glucose [5mM]
Glucose [5mM]
~kDa
44/42
ERK
Vinculin
125
Trametinib [1uM]
Uridine [1mM]
Glucose [5mM]
