# Supplementary material for: Uridine-derived ribose fuels glucose-restricted pancreatic cancer
Source: Nature. Author manuscript; Available in PMC 2024 Jun 1. (PMC10232363; doi:10.1038/s41586-023-06073-w)
Supplement: Supp Fig4 [file NIHMS1902848-supplement-Supp_Fig4.pptx]

## Slide 1
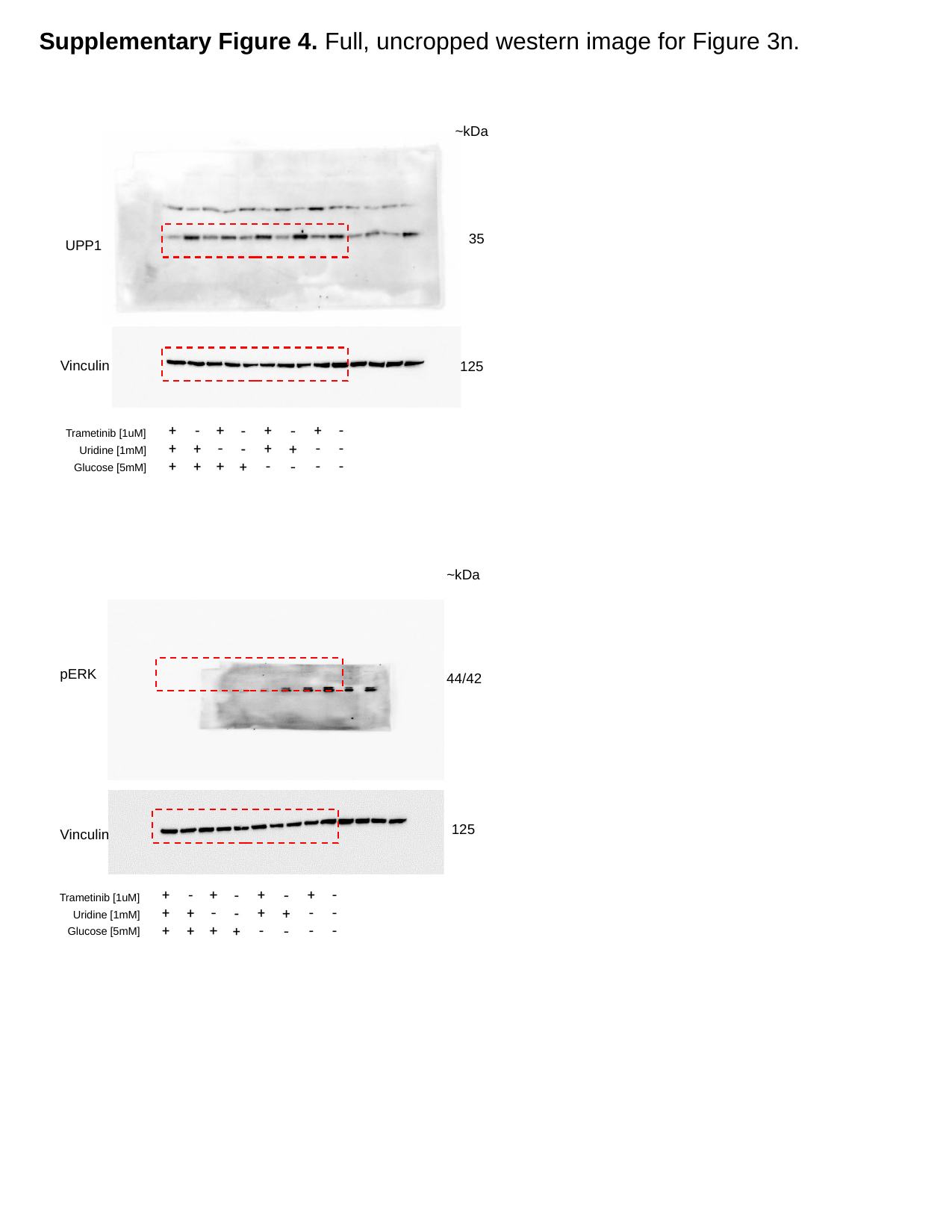

Supplementary Figure 4. Full, uncropped western image for Figure 3n.
~kDa
35
UPP1
Vinculin
125
Trametinib [1uM]
Uridine [1mM]
Glucose [5mM]
~kDa
pERK
44/42
125
Vinculin
Trametinib [1uM]
Uridine [1mM]
Glucose [5mM]
