# Supplementary material for: Uridine-derived ribose fuels glucose-restricted pancreatic cancer
Source: Nature. Author manuscript; Available in PMC 2024 Jun 1. (PMC10232363; doi:10.1038/s41586-023-06073-w)
Supplement: Supp Fig2 [file NIHMS1902848-supplement-Supp_Fig2.pptx]

## Slide 1
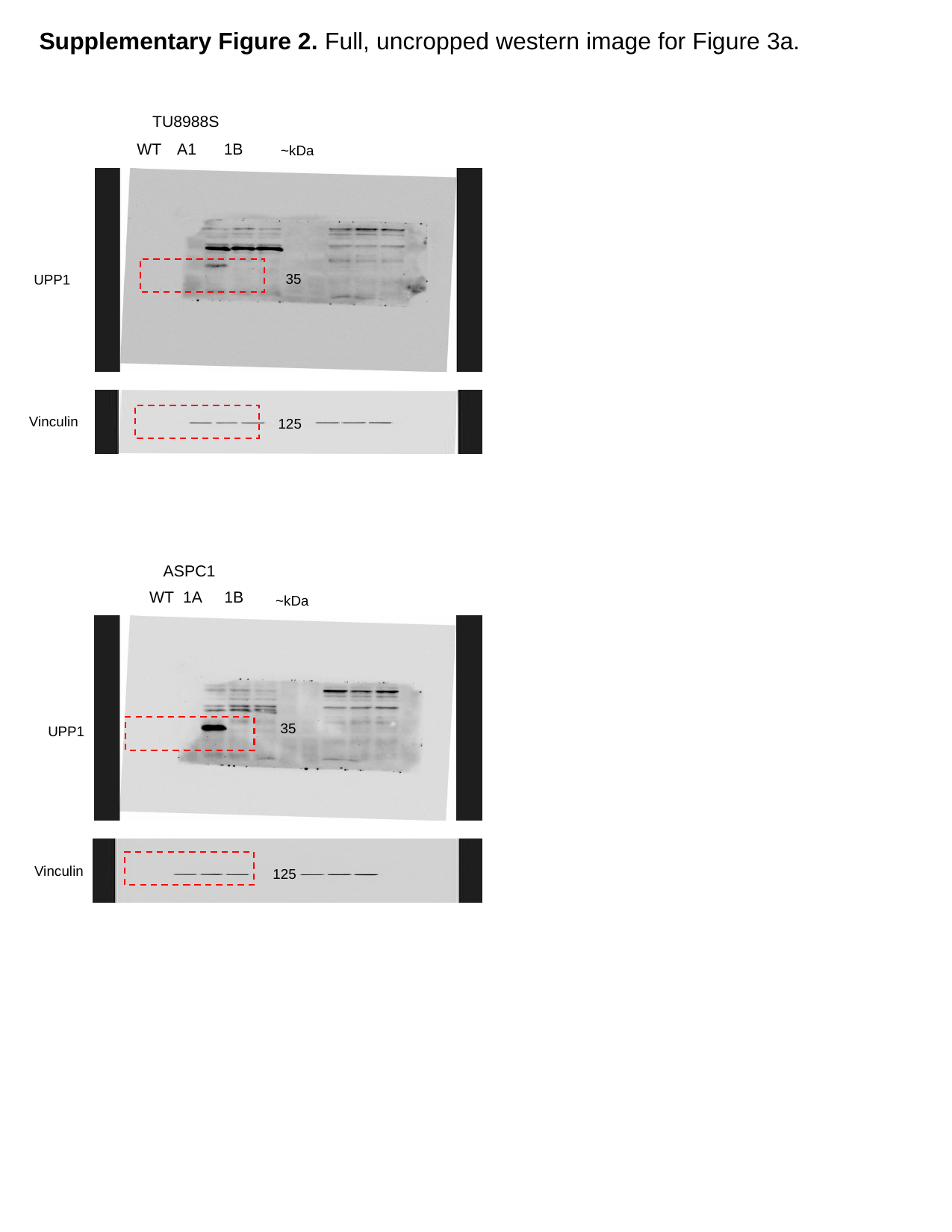

Supplementary Figure 2. Full, uncropped western image for Figure 3a.
TU8988S
WT
A1
1B
~kDa
35
UPP1
Vinculin
125
ASPC1
WT
1A
1B
~kDa
35
UPP1
Vinculin
125
