# Supplementary material for: Uridine-derived ribose fuels glucose-restricted pancreatic cancer
Source: Nature. Author manuscript; Available in PMC 2024 Jun 1. (PMC10232363; doi:10.1038/s41586-023-06073-w)
Supplement: Supp Fig1 [file NIHMS1902848-supplement-Supp_Fig1.pptx]

## Slide 1
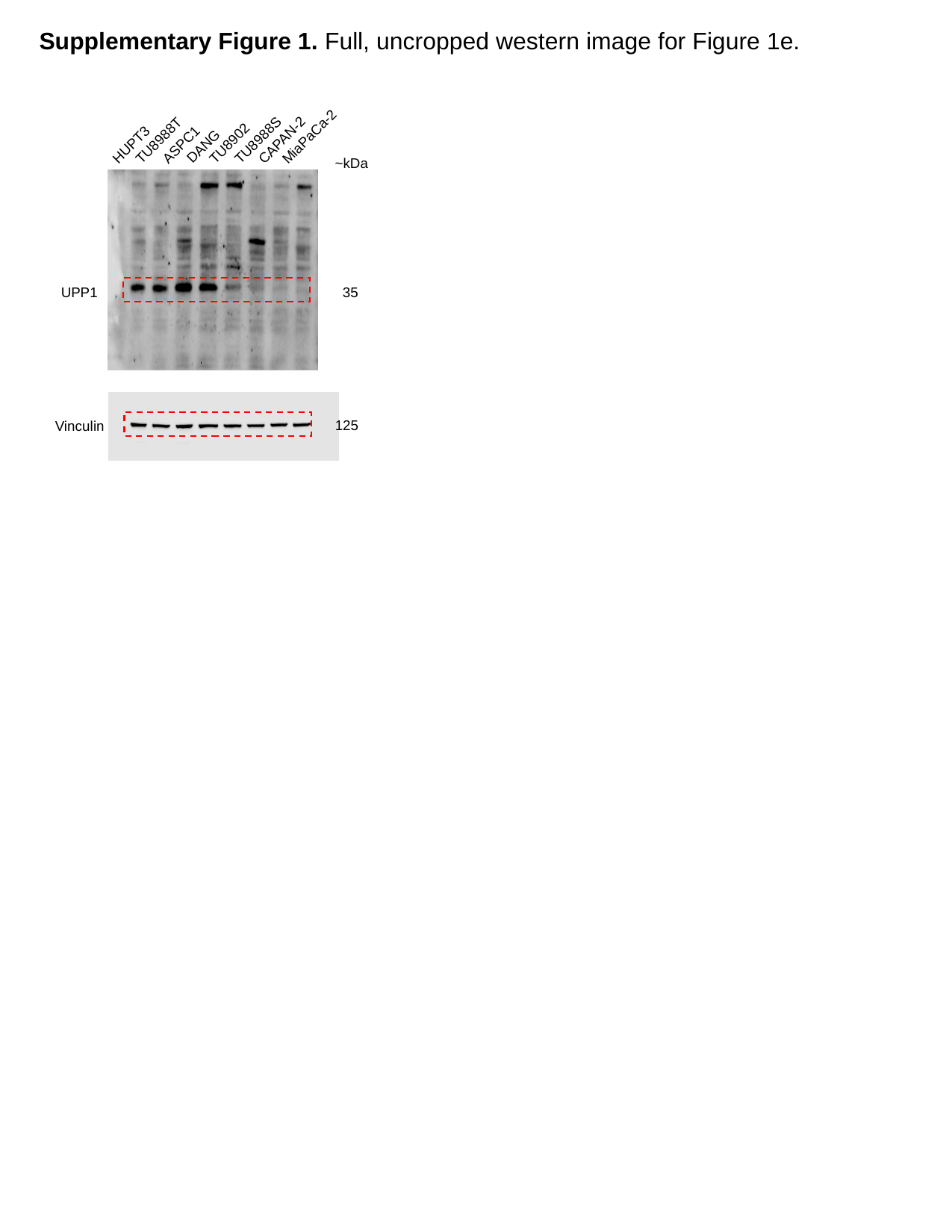

Supplementary Figure 1. Full, uncropped western image for Figure 1e.
MiaPaCa-2
CAPAN-2
TU8988S
TU8988T
TU8902
HUPT3
ASPC1
DANG
~kDa
UPP1
35
125
Vinculin
