# Supplementary material for: Uridine-derived ribose fuels glucose-restricted pancreatic cancer
Source: Nature. Author manuscript; Available in PMC 2024 Jun 1. (PMC10232363; doi:10.1038/s41586-023-06073-w)
Supplement: Supp Fig3 [file NIHMS1902848-supplement-Supp_Fig3.pptx]

## Slide 1
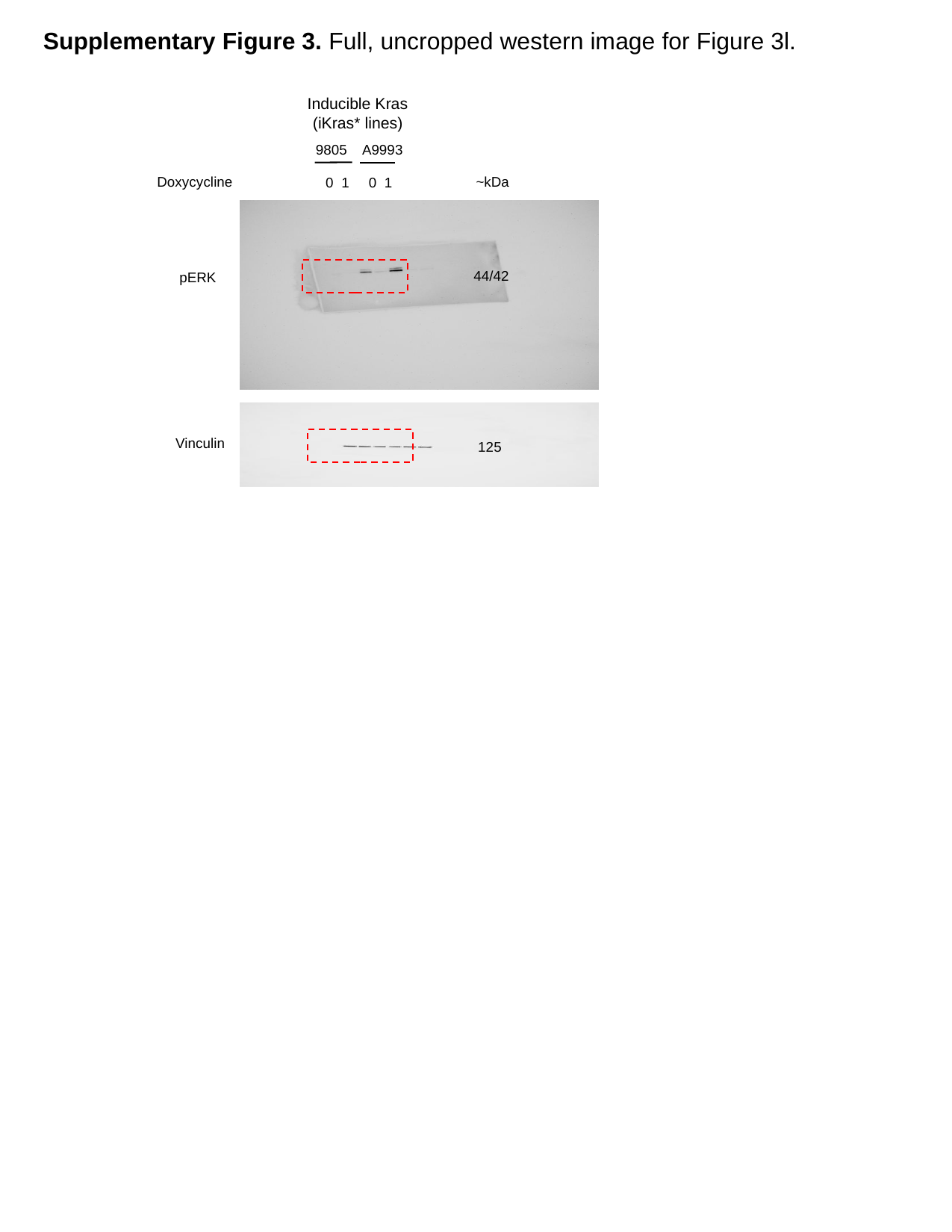

Supplementary Figure 3. Full, uncropped western image for Figure 3l.
Inducible Kras
(iKras* lines)
 9805 A9993
~kDa
Doxycycline
0 1 0 1
44/42
pERK
Vinculin
125
