# Supplementary material for: Uridine-derived ribose fuels glucose-restricted pancreatic cancer
Source: Nature. 2023 May 17;618(7963):151–8. doi: 10.1038/s41586-023-06073-w (PMC10232363; doi:10.1038/s41586-023-06073-w)
Supplement: Supplementary file 1 [file 41586_2023_6073_MOESM1_ESM.pdf]

nature portfolio

Corresponding author(s): Costas A. Lyssiotis

Last updated by author(s): Mar 27, 2023

Reporting Summary

Nature Portfolio wishes to improve the reproducibility of the work that we publish. This form provides structure for consistency and transparency in reporting. For further information on Nature Portfolio policies, see our Editorial Policies and the Editorial Policy Checklist.

Statistics

For all statistical analyses, confirm that the following items are present in the figure legend, table legend, main text, or Methods section.

n/a	Confirmed	
☐	☒	The exact sample size (*n*) for each experimental group/condition, given as a discrete number and unit of measurement
☐	☒	A statement on whether measurements were taken from distinct samples or whether the same sample was measured repeatedly
☐	☒	The statistical test(s) used AND whether they are one- or two-sided<br>*Only common tests should be described solely by name; describe more complex techniques in the Methods section.*
☒	☐	A description of all covariates tested
☐	☒	A description of any assumptions or corrections, such as tests of normality and adjustment for multiple comparisons
☐	☒	A full description of the statistical parameters including central tendency (e.g. means) or other basic estimates (e.g. regression coefficient) AND variation (e.g. standard deviation) or associated estimates of uncertainty (e.g. confidence intervals)
☐	☒	For null hypothesis testing, the test statistic (e.g. *F*, *t*, *r*) with confidence intervals, effect sizes, degrees of freedom and *P* value noted<br>*Give P values as exact values whenever suitable.*
☒	☐	For Bayesian analysis, information on the choice of priors and Markov chain Monte Carlo settings
☒	☐	For hierarchical and complex designs, identification of the appropriate level for tests and full reporting of outcomes
☒	☐	Estimates of effect sizes (e.g. Cohen's *d*, Pearson's *r*), indicating how they were calculated

Our web collection on statistics for biologists contains articles on many of the points above.

Software and code

Policy information about availability of computer code

Data collection	Biolog data was collected with the Omnilog instrument and software. Absorbance, luminescence, and fluorescence data was collected with SoftMax Pro 5.4.2 software. Agilent MassHunter software was used to collect and analyze metabolomics data. QuantStudio 3 and StepOne Plus version 2.3 software was used to collect real-time PCR data.
Data analysis	Biolog data was analyzed using the R opm package version 1.3.77. Immunohistochemistry data was analyzed with CellSens standard software and FIJI/Image J (version 1.53c). Antigen expression was scored using Definiens Test Studio Software (Definiens). Metabolomics data were analyzed with Agilent MassHunter Workstation Quantitative Analysis for QQQ Version 10.1, Build 10.1.733.0. Isotope enrichment data were analyzed with Agilent MassHunter Workstation Profinder Version 10.0, Build 10.0.10062.0 and Skyline Daily (version 22.2.1.256). Pathway analyses were performed using DAVID functional annotation platform (https://david.ncifcrf.gov/, version 6.8) or the gene set enrichment analysis (GSEA, version 4.0.3). Statistics were performed either with GraphPad Prism 8 (GraphPad Software Inc.) or using R version 3.5.2. For data analysis and visualization in R, packages (with versions) used include dplyr (0.8.3), ggplot2 (3.3.5), gplots (3.0.1, heatmap.2 function), ComplexHeatmap (2.3.5), tidyverse (1.3.0) and VennDiagram (1.6.20).

For manuscripts utilizing custom algorithms or software that are central to the research but not yet described in published literature, software must be made available to editors and reviewers. We strongly encourage code deposition in a community repository (e.g. GitHub). See the Nature Portfolio guidelines for submitting code & software for further information.

Data

Policy information about availability of data

All manuscripts must include a data availability statement. This statement should provide the following information, where applicable:

- Accession codes, unique identifiers, or web links for publicly available datasets
- A description of any restrictions on data availability
- For clinical datasets or third party data, please ensure that the statement adheres to our policy

Human PDA data used in this study are publicly accessible under the accession numbers indicated in the Methods section “PDA dataset analysis”, “pan-cancer data analysis” and GSE36133 (for CCLE data). Other experimental data (e.g., the nutrient profiling and metabolomics data) have been summarized and presented in this study. The gene microarrays were obtained from NCBI gene expression omnibus, the ICGC-AU microarray data (release_28) was downloaded from https://dcc.icgc.org/projects/ along with the associated clinical data and had no embargo (March 2020) and the TCGA data was downloaded from CBioPortal (https://www.cbioportal.org/). CCLE protein data was accessed via DepMap portal (https://depmap.org/portal/). Human Protein Atlas data is available from: URL for ‘Normal’ - https://www.proteinatlas.org/ENSG00000183696-UPP1/tissue/pancreas; PDA – https://www.proteinatlas.org/ENSG00000183696-UPP1/pathology/pancreatic+cancer#img. The accompanying source data are provided as Supplemental Tables. All other data that support the findings of this study are available from the corresponding authors upon request.

Human research participants

Policy information about studies involving human research participants and Sex and Gender in Research.

Reporting on sex and gender	Sex and gender were not provided or used in the selection of slides for histological analysis.
Population characteristics	Human samples were from patients diagnosed with pancreatic cancer at the University of Michigan between 2021-2022 that were eligible for resection. No other population characteristics were considered in the analysis.
Recruitment	Deidentified samples from the tissue repository supported by IRB (HUM00025339) were used for histological analysis.
Ethics oversight	The collection of patient-derived samples was approved by the Institutional Review Board at the University of Michigan (IRB number: HUM00098128).

Note that full information on the approval of the study protocol must also be provided in the manuscript.

Field-specific reporting

Please select the one below that is the best fit for your research. If you are not sure, read the appropriate sections before making your selection.

☒ Life sciences ☐ Behavioural & social sciences ☐ Ecological, evolutionary & environmental sciences

For a reference copy of the document with all sections, see nature.com/documents/nr-reporting-summary-flat.pdf

Life sciences study design

All studies must disclose on these points even when the disclosure is negative.

Sample size	No sample size calculation was performed. Instead, sample sizes were chosen based on standard experimental group sizes to achieve acceptable power taking into account the increased variability of animal models (3-4 biological replicates for in vitro experiments, and 7-8 biological replicates for in vivo experiments). For metabolomics, isotope tracing, and qPCR experiments, sizes of 3-4 biological replicates were chosen based on the variability observed in pilot studies and in previous publications (citation below). For western blotting, generally one biological replicate representative of >=2 experiments was shown. For in vitro metabolic assays (e.g., MTT, Biolog, CellTiter Glo) generally 4 biological replicates were included in the experimental design based on the variation observed in earlier repetitions of the same experiment and in previous publications (citation below).<br>Citation: Nwosu, Z. C. et al. Severe metabolic alterations in liver cancer lead to ERK pathway activation and drug resistance. EBioMedicine 54, (2020).
Data exclusions	No data were excluded from the analyses.
Replication	All experiments were successfully reproduced a minimum of two times with at least 3 biological replicates, with the exception of the metabolomics studies which were typically only run once using samples prepared from biological replicates (n=3).
Randomization	Experimental groups with mice were assigned randomly. Experimental groups for in vitro studies were not randomized as the cells are taken from a population assumed to be homogeneous and clonally identical. Experimental groups were not randomized for histological analysis of patient-derived biospecimens as the groups being compared (tumor and adjacent normal tissue) are paired.
Blinding	Blinding was not performed for in vitro or in vivo analyses as the measurements taken are objective.

Reporting for specific materials, systems and methods

We require information from authors about some types of materials, experimental systems and methods used in many studies. Here, indicate whether each material, system or method listed is relevant to your study. If you are not sure if a list item applies to your research, read the appropriate section before selecting a response.

Materials & experimental systems

n/a	Involved in the study
☐	☒ Antibodies
☐	☒ Eukaryotic cell lines
☒	☐ Palaeontology and archaeology
☐	☒ Animals and other organisms
☒	☐ Clinical data
☒	☐ Dual use research of concern

Methods

n/a	Involved in the study
☒	☐ ChIP-seq
☒	☐ Flow cytometry
☒	☐ MRI-based neuroimaging

Antibodies

Antibodies used	For western blotting, the following primary antibodies were used in this study and at 1:1000 dilution: anti-UPP1 (Sigma-Aldrich, # HPA055394), anti-c-MYC (Cell Signaling, # 5605S), anti-pERK (Cell Signaling, # 9106L), anti-ERK (Cell Signaling, # 9102S), anti-PGM2 (Invitrogen, # PA5-31378), and anti-Vinculin (Cell Signaling, # 13901S). The following secondary antibodies were used: anti-rabbit-HRP (Cell Signaling, # 7074S), and anti-mouse-HRP (Cell Signaling, # 7076P2).<br>For immunohistochemistry, the following primary antibodies were used in this study: Rabbit anti-UPP1 (1:200; Sigma-Aldrich, # HPA055394), Anti-Cd31 monoclonal antibody (1:75, clone SZ31, # DIA-310, Dianova), Anti-Cd8 monoclonal antibody (1:200, clone -4SM15, # 14-0808, eBioscience), Anti-Cd3 polyclonal antibody (1:400, # ab5690, Abcam), and anti-F4/80 monoclonal antibody (1:100, Clone-A3-1, # MCA497G, Bio-Rad). The following secondary antibodies were used in this study: biotinylated secondary antibody (Horse anti-Rabbit; 1:500; Vector Labs, # BA-1100).<br>For RNAscope, primary antibody for panCK (Mouse anti-cytokeratin, pan reactive; 1:100; BioLegend; # 628602) was used.
Validation	anti-UPP1 (Sigma, HPA055394) - Figure 1e, Figure 3a<br>anti-c-MYC (Cell Signaling, 5605S) - Extended Figure 9a, 9b<br>anti-pERK (Cell Signaling, 9106L) - Extended Figure 8c<br>anti-ERK (Cell Signaling, 9102S) - Extended Figure 8c<br>anti-PGM2 (Invitrogen, PA5-31378) - Extended Figure 4i<br>anti-Vinculin (Cell Signaling, 13901S) - Figure 1e, Extended Figure 8c<br>anti-cytokeratin (BioLegend, 628602) - Figure 3g, Extended Figure 7a

Eukaryotic cell lines

Policy information about cell lines and Sex and Gender in Research

Cell line source(s)	The panel of PDA cell lines, HPNE, A549, HT1080, HCT116, and U2OS, were purchased from the American Type Culture Collection (ATCC) or the German Collection of Microorganisms (DSMZ). The hPSC cell line and the mouse cell lines KPC 7940b and MT3-2D were generously provided under MTA by Rosa Hwang (MD Anderson Cancer Center), Gregory Beatty (University of Pennsylvania), and David Tuveson (Cold Spring Harbor Labs), respectively. iKras cell lines A9993 and iKRAS 9805 were derived as previously described (Collins et al., 2012, JCI, PMID: 22232209).
Authentication	All cell lines were authenticated with STR genetic testing.
Mycoplasma contamination	Cell lines regularly tested negative for mycoplasma using the MycoAlert kit (Lonza).
Commonly misidentified lines<br>(See ICLAC register)	None used.

Animals and other research organisms

Policy information about studies involving animals; ARRIVE guidelines recommended for reporting animal research, and Sex and Gender in Research

Laboratory animals	6-8 weeks old and 8-12 weeks old C57BL/6J mice (The Jackson Laboratory (Strain # 000664); ~6 weeks old C57BL/6NCrl mice (Charles River Laboratories (Strain Code 027).
Wild animals	None used.
Reporting on sex	Studies were carried out with a mix of male and female mice or only female mice. No specific method was used for assigning sex and sex-based difference was not observed.

Field-collected samples	None used.
Ethics oversight	Animal studies were performed at the University of Michigan (UM), the Institute of Cancer Research (ICR), and the University of Chicago (UChicago) according to approved protocols. UM Institutional Animal Care and Use Committee (IACUC) PRO00010606; ICR studies conformed to UK Home Office Regulations under the Animals Scientific Procedures Act 1986 and national guidelines (Project licence-P0A54750A protocol 5); UChicago IACUC Protocol #72587.

Note that full information on the approval of the study protocol must also be provided in the manuscript.
